# Castleman’s disease in the rheumatological practice

**DOI:** 10.1007/s00393-024-01560-5

**Published:** 2024-08-22

**Authors:** M. Schmalzing, O. Sander, M. Seidl, R. Marks, N. Blank, I. Kötter, M. Tiemann, M. Backhaus, B. Manger, K. Hübel, U. Müller-Ladner, J. Henes

**Affiliations:** 1 Department of Medicine and Polyclinic II, University Hospital Würzburg, Oberdürrbacher Straße 6, 97080 Würzburg, Germany; 2 Department of Rheumatology, University Hospital Düsseldorf, Düsseldorf, Germany; 3 Institute of Pathology, University Hospital Düsseldorf, Düsseldorf, Germany; 4 Department of Internal Medicine I, University Hospital Freiburg, Freiburg, Germany; 5 University Hospital Heidelberg, Heidelberg, Germany; 6 Department of Rheumatology and Immunology, Hospital Bad Bramstedt, Bad Bramstedt, Germany; 7 Rheumatology, University Hospital Hamburg-Eppendorf, Hamburg, Germany; 8 Institute of HematoPathology Hamburg, Hamburg, Germany; 9 Dept. of Internal Medicine—Rheumatology and Clinical Immunology, Park-Klinik Weissensee (Berlin), Berlin, Germany; 10 Department of Medicine 3—Rheumatology and Immunology, University Hospital Erlangen, Erlangen, Germany; 11 Department of Internal Medicine, University Hospital Cologne, Cologne, Germany; 12 Department of Rheumatology and Clinical Immunology, Kerckhoff Klinik Bad Nauheim, Bad Nauheim, Germany; 13 Department of Medicine II, University Hospital Tübingen, Tübingen, Germany

Due to the heterogeneous clinical symptoms, a significant proportion of patients with Castleman’s disease (CD) are likely to be misdiagnosed or remain unrecognized. The overlaps that exist with malignant, infectious, or autoimmune diseases and syndromes also contribute to this. In particular, idiopathic multicentric CD (iMCD), as a potentially life-threatening systemic disease, represents an important differential diagnosis in rheumatological practice.

Castleman’s disease (CD) was originally described by the American pathologist Benjamin Castleman, who initially characterized the clinical features in 1954 in patients with solitary mediastinal lymph node hyperplasia as a “peculiar form of lymph-node hyperplasia” [1]. The following case reports illustrate why the differential diagnostic distinction from autoimmune diseases of rheumatological origin is often particularly challenging.

## Case report 1

**Fig. 1:**
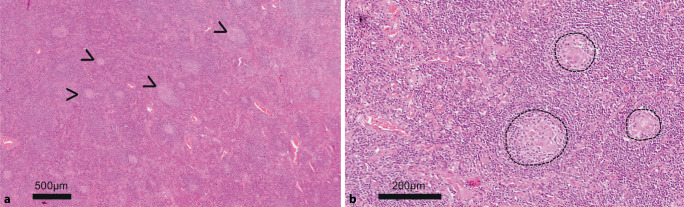
Histology for case report 1 (5× and 20× magnification for **a** and **b**, respectively). **a** Overview magnification of the axillary lymph node with secondary lymphofollicular hyperplasia (secondary follicles marked by an* arrowhead *as an example). The overall very small secondary follicles are striking. **b** Regressive, hyalinized germinal centers (*dashed line*) with an at least partially layered mantle zone (germinal center *bottom left*)

A 62-year-old female patient was referred to the rheumatology department of a university hospital for the first time with clinical suspicion of connective tissue disease. The patient presented with truncal erythema and a long-standing, recently regressed lymphadenopathy. Furthermore, the patient had a history of Raynaud’s syndrome, pulmonary fibrosis, and pulmonary arterial hypertension (PAH) of World Health Organization (WHO) functional class II. The current laboratory tests focused on a pronounced polyclonal gammopathy (after exclusion of amyloidosis). Serologically, there were also high-titer ANA levels (titer of 1:20,480 with a speckled ANA pattern) and positive antibody findings against RNP, Sm, SSA, SSB, and dsDNA. Retrospectively, elevated serum total protein (9.4 g/dL) and mild anemia (10.3 g/dL) had been reported 8 years earlier, and marked polyclonal hypergammaglobulinemia (IgG > 5 g/dL) had been reported more than 1 year previously. C‑reactive protein (CRP) was either normal or elevated up to 11 mg/dL during the course. Furthermore, it was known from the past medical history that the patient had already been hospitalized several times for episodes of fever, elevated inflammatory markers, exanthema, and other autoimmune phenomena. Usually, an infection was treated first, with an improvement of symptoms reportedly occurring under antibiosis. Relevant therapeutic effects under steroids were observed only under very high doses. During a myositis workup 6 months previously, a left axillary lymph node biopsy was performed, initially found to be “nonspecific lymphadenitis.” One month later, subsequent workup was performed by a lymphoma center of reference. According to this, the histopathological findings corresponded to MCD with secondary lymphofollicular hyperplasia (Fig. 1).

## Case report 2

**Fig. 2:**
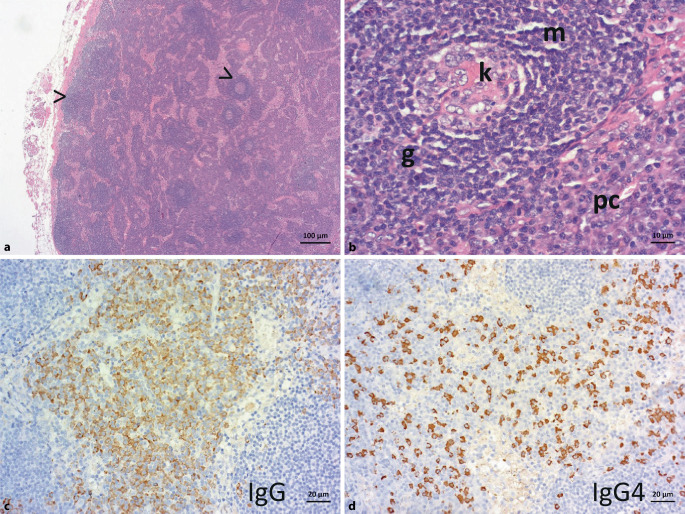
Histology for case report 2 (5×, 40×, 10×, 20×  magnification for **a–c** and **d**, respectively). **a** Overview magnification of the mediastinal lymph node with preserved compartments (follicles are exemplarily marked by an *arrowhead*; *left* is without and *right* is with a germinal center). **b** Typical “lollipop” pattern of the hyaline vascular transformed regressive germinal center (*k*) due to the prominent vessel (*g*) and the onion skin-like structured mantle zone (*m*); adjacent to this, perisinusoidal/perivenular increased plasma cells (*pc*). **c** Condensed follicular dendritic cells within the germinal centers, **d** with an overall significantly increased plasma cell count; there are also increased IgG 4-positive plasma cells, but without eosinophilia, fibrosis, or obliterative phlebitis, thus histologically atypical for IgG 4-associated disease. The proliferation of plasma cells is indicated serologically by hypergammaglobulinemia

A 22-year-old male patient presented to the emergency department with chest pain and an unclear constellation of infections. Initial diagnosis revealed acute renal failure, partial respiratory insufficiency, and multilocular lymphadenopathy (hilar, mesenteric, retroperitoneal, and para-iliac) as well as thymic hyperplasia. Repeated pathogen diagnostics remained negative during the course. Due to deterioration of the general condition with bilateral pleural effusions and ascites within 3 weeks, the patient was admitted to the intensive care unit. Histological examination showed chronic fibrosing mediastinitis and granulating pleuritis, and histological bone marrow examination revealed a trilinear increase in hematopoiesis with a leading increase in megakaryocytes. Following a renewed intervention for mediastinal lymph node bulks, the asservation of a whole lymph node was performed. Histopathologic examination was typical for MCD (Fig. 2).

## Systematology

**Fig. 3:**
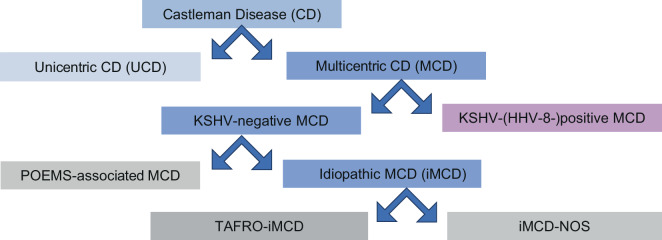
Clinical classification of Castleman’s disease according to lymph node involvement and clinical presentation (modified according to [3])

Based on clinical presentation, multicentric and unicentric Castleman’s disease are differentiated from one another. Multicentric Castleman’s disease (MCD), unlike unicentric Castleman’s disease (UCD), is not a localized and easy-to-treat hyperplasia of lymphoid tissue: the multilocular lymphadenopathy in MCD may be associated with severe systemic progressive disease and may be lethal if untreated [2]. From an etiopathogenetic point of view, a further subdivision into HHV‑8 (human herpesvirus 8; also Kaposi’s sarcoma-associated herpesvirus, KSHV)-associated or HHV-8-negative MCD of unknown cause (idiopathic MCD, iMCD) is important [3]. HHV8-associated MCD is most commonly observed in HIV-infected or immunocompromised individuals [2, 3]. In the iMCD-TAFRO subtype first described in Japan, the recently established acronym “TAFRO” stands for thrombocytopenia, ascites, fever, reticular fibrosis in the bone marrow, and organomegaly [4]. However, patients with “not otherwise specified” iMCD (iMCD-NOS) do not have TAFRO syndrome [5]. Rarely, iMCD is associated with a POEMS syndrome, consisting of polyneuropathy, organomegaly, endocrinopathy, monoclonal gammopathy, and skin changes (POEMS-associated iMCD; Fig. 3).

## Epidemiology

Given the rarity of the disease, the data regarding the frequency of Castleman’s disease are insufficient. The limitations in the ability to evaluate the disease pattern can also be attributed to the diagnostic criteria and specific ICD-10 codes that were introduced just a few years ago (2016, 2017) [2, 5]. However, both the incidence and prevalence of Castleman’s disease are likely to be underestimated [6]. According to an analysis of US health insurance data carried out for the first time according to the newer diagnostic criteria, around 3326 patients with CD were prevalent in 2017 and around 5282 patients in 2018. The incidence based on the year 2017 (2018) was calculated as 3.4 (3.1) and the prevalence as 6.9 (9.7) iMCD cases per million individuals, although an underreported prevalence can be assumed. In principle, Castleman’s disease can affect any age group. The median age at initial diagnosis of MCD is in the fifth to sixth decade of life, and for UCD is 40 years. MCD tends to affect men more often than women [7].

## Pathogenesis

### Unicentric Castleman’s disease

The pathogenesis of UCD is only incompletely understood, although recent evidence suggests that it may be a neoplastic process of lymph node stromal cells (LNSCs) involving follicular dendritic (reticulum) cells (FDC) [7, 8]. In 17% of the UCD cases studied, mutations in *PDGFRB* (platelet-derived growth factor receptor-β) were identified [9]. An association with viral infections has not been found [5].

### Multicentric Castleman’s disease

To date, the pathomechanism is best understood in HHV-8-positive MCD (KSHV-MCD), which is observed in both HIV-positive and HIV-negative HHV-8-positive patients and leads to polyclonal lymphoproliferation. Up to 30% of B lymphocytes in the mantle zone of lymphoid follicles become infected with HHV‑8, almost exclusively affecting B cells expressing IgM-lambda light chains. Viral genes are expressed during viral latency (e.g., LANA) as well as during lytic viral replication (e.g., viral interleukin‑6, vIL-6) and can be detected in KSHV-MCD. The viral protein vIL‑6, which is homologous to human IL‑6 and is mainly observed in plasma blasts surrounding lymphoid follicles, is considered to be particularly characteristic. In addition to excessive release of IL‑6 and vIL‑6, other inflammatory cytokines such as IL-10, IL-1β, and TNF are shown to be dysregulated (cytokine storm) in KSHV-MCD [2].

The etiology of iMCD has not been definitively determined, with at least one of the following hypothetical pathomechanisms being discussed: autoimmunity, autoinflammation, neoplastic processes, and/or as yet unknown viral infections of pathogenetic relevance [3]. It is conceivable that different pathomechanisms lead to the cytokine and chemokine storm or cause a similar clinical phenotype. In addition to IL‑6, other proinflammatory cytokines and chemokines can be involved in the pathogenesis, such as VEGF, IL‑1, IL‑8, CXCL13, or TNFα [2, 3, 10]. In addition, T cell activation and activation of the mTOR (mammalian target of rapamycin) or JAK-STAT3 (Janus kinase signal transducer and activator of transcription 3) or type I interferon signaling pathways could play a role. In the process of (de)activation of certain genes, the possibility of increased inflammasome activity and thus autoinflammatory genesis has also been discussed [2].

## Clinical presentation

The localized form of UCD usually presents as visible or palpable lymphadenopathy. UCD can become symptomatic when neighboring structures are displaced. In contrast, constitutional symptoms such as fatigue or weight loss are rare and usually absent in UCD [2]. MCD, on the other hand, is rarely asymptomatic. The disease shows a relapsing course, with patients often being seriously ill during the relapse. Often pronounced B symptoms with fever, night sweats, and weight loss can be associated with splenomegaly, very often also with hepatomegaly and anasarca (massive generalized edema due to hypoalbuminemia), and non-specifically elevated biochemical inflammatory markers (especially ESR and CRP), anemia, and hypergammaglobulinemia can the indicate the presence of the disease. Renal involvement with consecutive renal failure and proteinuria is mainly observed in HHV-8-negative iMCD patients [11]. In a variety of disease processes, hypergammaglobulinemia can be the result of polyclonal B cell activation. When interpreting laboratory serological findings, it must be taken into account that hypergammaglobulinemia can be associated with false-positive immune serologies (e.g., detection of several autoantibodies) [12]. The clinical symptoms tend to be more pronounced in HHV-8-positive MCD patients than in patients with iMCD, as the data from the two largest case series and meta-analysis suggest (Table 1; [11, 13]). Secondary amyloidosis and membranoproliferative glomerulonephritides can be observed due to kidney involvement, while pulmonary symptoms can include lymphoid interstitial pneumonitis (LIP), a restrictive lung disease, or bronchiolitis obliterans. Potential skin changes to be considered are, among others, rash, hyperpigmentation, tardive hemangioma (“cherry angioma”), paraneoplastic pemphigus, and Kaposi’s sarcoma [7]. Furthermore, many patients report fatigue, loss of appetite, and nausea [14]. In rare single cases, patients with iMCD have been described who developed rheumatoid arthritis (RA)-like active arthritis. Accordingly, T cell activation could also play a pathophysiologically relevant role in the development of arthritis and lead to the comorbid occurrence of these RA-like symptoms in iMCD [15].

**Table 1:** Selected common clinical findings in HHV-8-positive vs. HHV-8-negative MCD [11, 13]

	HHV-8-positive (%)	HHV-8-negative (%)
Fever	88	73
Splenomegaly	78	41–78
Edema	40	23–78
Lung involvement	26	48
Skin changes	13	22
Renal involvement	6.5	41
Hemophagocytosis	38	4

## Diagnostics and histopathology

**Fig. 4:**
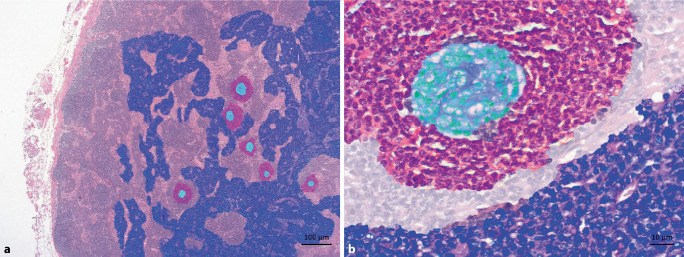
False-color imaging for case report 2. **a** Overview magnification to highlight the clear plasma cell proliferation (shown in *purple*; *turquoise *germinal centers, *magenta *mantle zone). **b** Higher magnification: plasma cell location is physiologically perisinusoidal/perivenular. Afferent lymphatic vessels drain into the lymph node sinuses, i.e., antigens arriving from the periphery first come into contact with antibodies of the already established plasma cells. Class-switched plasma cells, in turn, originate from the germinal center response (*turquoise*) as a selection outcome in competition for the antigen, which is stored on follicular dendritic cells (FDC) in the germinal center in an immune complex-bound manner (higher-affinity antibodies displace lower-affinity ones in the course of the immune response). Naive and memory B cells of the mantle zone (*magenta*) may enter the germinal center response

**Fig. 5:**
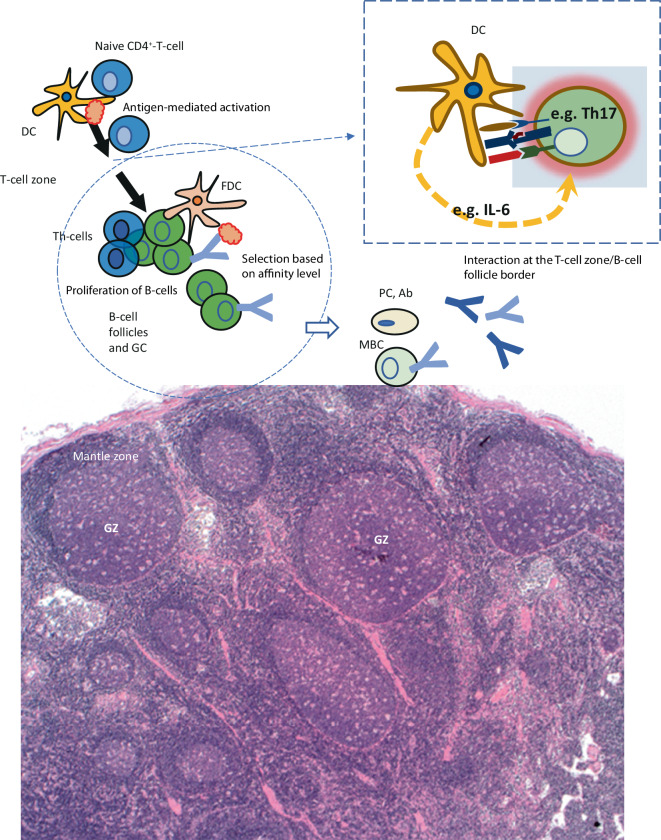
Schematically simplified presentation of the germinal center reaction. *Ab* antibodies, *DC* dendritic cell, *FDC* follicular DC, *GC* germinal center, *MBC* memory B cells, *PC* antibody-producing long-lived plasma cells

The example patient case 2 makes CD histopathology understandable using a false-color presentation: the patient had regressive germinal centers, with a significantly reduced B cell proportion in condensed, immune complex-loaded follicular dendritic cells (Fig. 4). Accordingly, in CD, regressive germinal centers can be the result of significant plasma cell expansion and excessive antibody production, which makes antigen access increasingly difficult for germinal center B cells (Fig. 5).

**Fig. 6:**
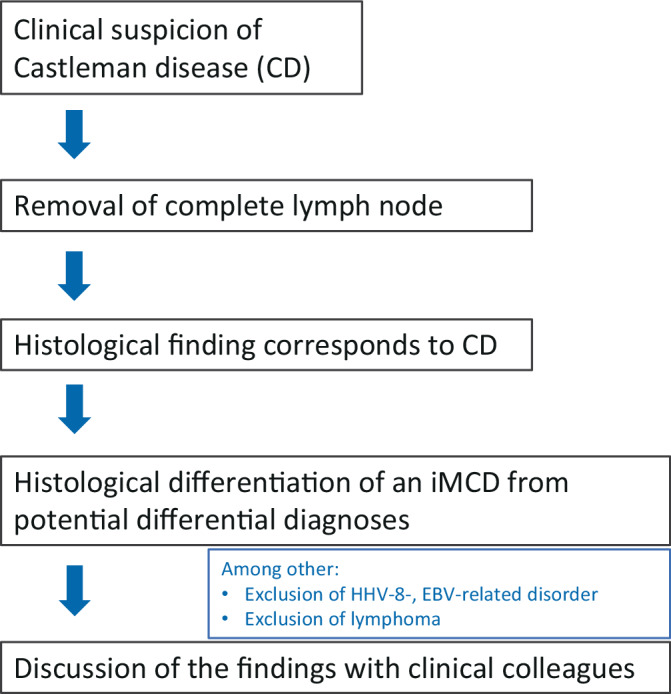
In case of clinical suspicion of Castleman’s disease: procedure from the pathologist’s point of view

From the pathologist’s perspective, two prerequisites are essential for an early diagnosis: firstly, to consider the differential diagnoses of CD, and secondly, to remove a complete lymph node for optimal histological assessment. A puncture is not sufficient to be able to achieve a reliable CD diagnosis. The main pathological changes include follicular hyperplasia with regressive, partly hyaline vascular transformed germinal centers and plasma cell expansion and can already be detected morphologically by H&E staining [3, 11]. If the histological findings are consistent with a CD diagnosis, a histological distinction from iMCD and the most important differential diagnoses must still be made (Fig. 6). Difficulties can arise especially in the early phase of the disease, when regressive germinal centers are sparse. There may be a risk of confusion due to reactively increased immunoblasts and/or oligoclonal T cell expansions, which can also occur in CD and must be clarified with regard to infectious genesis or lymphomas to differentiate from iMCD [3, 16, 17]. In particular, plasma cell-maturing lymphomas (exclusion via light chain immunohistochemistry and destroyed lymph node architecture) as well as HIV-associated lymphadenopathies should be listed as histomorphological differential diagnoses of iMCD, whereby the differentiation from various KSHV-associated diseases can be challenging, especially in HHV-8-positive MCD (exclusion via Kaposiform vascular transformation and immunohistochemistry; caveat: e.g., monotypic λ‑light chain expression by plasma blasts in HHV-8-associated germinotropic lymphoproliferative disease). The exclusion of diseases with Castleman-like histology is also part of the international consensus criteria for iMCD diagnosis (Table 2; [3]). Before the extirpation and histological workup of a lymph node, CT-based imaging should also be considered as a useful screening tool in the area of the neck, thorax, and abdomen (if necessary, by PET-CT). Lymph node sampling should be carried out at the latest in cases of treatment-refractory lymphadenopathy. If possible, the removal should ideally take place before initiating immunomodulatory therapy, such as steroid administration. Glucocorticoids in particular have a broadly suppressive effect on cells of the innate and adaptive immune system [18] and, according to current guidelines (as of May 2023), are an integral part of numerous lymphoma therapies, including for plasma cell neoplasms. It can therefore be assumed that glucocorticoids have an inhibitory effect on active, proliferating components of CD such as plasma cells and germinal centers, especially in the early stages of the germinal center reaction via inhibition of T follicular helper cells [19]. Whether this in turn leads to increased regressive germinal centers has not yet been systematically investigated. However, germinal centers that have already become regressive are more likely to persist under steroid administration due to their hypocellularity. From a histopathological point of view, an attenuated underlying disease is more difficult to diagnose, and it is difficult to estimate the possible additional superimposition of infections due to increased vulnerability under glucocorticoid therapy.

**Table 2:** International diagnostic consensus criteria for iMCD [3]

*Major criteria (both required)*
Lymph node with typical histology and
Enlargement of lymph nodes (>1 cm) in at least 2 node stations
*Minor criteria (at least 2 of 11 criteria with at least 1 laboratory criterion):*
*Laboratory*
↑CRP (> 10 mg/L) or ESR (> 15 mm/h)
Anemia (Hb < 12.5 g/dL for males, Hb < 11.5 g/dL for females)
Thrombocytopenia (platelet count < 150/pL) or thrombocytosis (thrombocytes > 400/pL)
Hypalbuminemia (albumin < 3.5 g/dL)
Renal dysfunction (eGFR < 60 mL/min/1.73 m^2^) or proteinuria (total protein 150 mg/24 h or 10 mg/100 mL)
Polyclonal hypergammaglobulinemia (> 1700 mg/dL)
*Clinical*
B symptoms (night sweats, fever, weight loss, or fatigue)
Splenomegaly and/or hepatomegaly
Edema, anasarca, ascites, pleural effusion
Eruptive hemangiomatosis or purple papules
Lymphocytic interstitial pneumonitis
*Exclusion criteria*
Infection-related disorders: HHV‑8 (blood PCR, positive LANA‑1 staining via immunochemistry), clinical EBV-lymphoproliferative disorders, adenopathy caused by HIV, CMV, toxoplasmosis, active tuberculosis
Autoimmune/autoinflammatory diseases (detection of autoimmune antibodies alone is not an exclusion criterion for iMCD): systemic lupus erythematosus, rheumatoid arthritis, adult-onset Still’s disease, juvenile idiopathic arthritis, autoimmune lymphoproliferative syndrome
Malignant/lymphoproliferative disorders (diagnosed before or at the same time as iMCD to be exclusionary): Hodgkin’s and non-Hodgkin’s lymphoma, multiple myeloma, primary lymph node plasmocytoma, FDC sarcoma, POEMS syndrome

## Differential diagnosis of autoimmune diseases

**Fig. 7:**
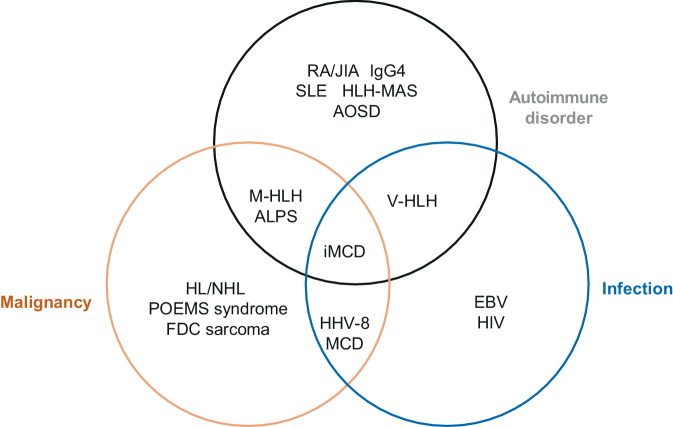
Spectrum of possible differential diagnoses of autoimmune, malignant, and infection-related diseases in iMCD. *ALPS* autoimmune lymphoproliferative syndrome*; AOSD* adult-onset Still’s disease; *EBV* Epstein–Barr virus; *FDC* follicular dendritic cell; *HHV‑8* human herpesvirus 8; *HLH* hemophagocytic lymphohistiocytosis; *M‑HLH* malignancy-associated HLH; *V‑HLH* virus-associated HLH; *HLH-MAS* HLH macrophage activation syndrome; *POEMS* polyneuropathy, organomegaly, endocrinopathy, monoclonal gammopathy, skin lesions; *RA* rheumatoid arthritis; *JIA* juvenile idiopathic arthritis; *SLE* systemic lupus erythematosus (according to Fajgenbaum et al. [3])

In contrast to UCD, the clinical picture of patients with MCD is often characterized by non-specific symptoms such as fever, night sweats, and weight loss (B symptoms) and a pronounced general feeling of malaise, which indicate a severe, highly inflammatory systemic disease. From a clinical point of view, the spectrum of autoimmune diseases in iMCD that should be considered in the differential diagnosis includes systemic lupus erythematosus (SLE); Sjögren’s syndrome; rheumatoid arthritis (RA); adult-onset Still’s disease (AOSD); juvenile idiopathic arthritis (JIA); autoimmune lymphoproliferative syndrome (ALPS); IgG 4-related diseases (IgG 4-RD); and hyperinflammatory syndromes such as macrophage-activation syndrome (MAS-HLH), hemophagocytic lymphohistiocytosis (HLH), VEXAS syndrome (VEXAS = vacuoles, E1 enzyme, X‑linked, autoinflammatory, somatic), and fulminant sarcoidosis. Furthermore, diseases of malignant origin, infectious diseases (acute infections with the Epstein–Barr virus [EBV] or HIV), and overlapping syndromes (overlap syndromes) must be considered in the differential diagnosis (Fig. 7; [3]). In addition, the COVID-19 pandemic has expanded the spectrum of infectious diseases relevant for differential diagnosis to include SARS-CoV‑2 (SARS-CoV-2-induced cytokine dysregulation).

## Systemic lupus and Sjögren’s syndrome

As case report 1 illustrates, the exclusion of infections and tumors, especially in the case of a prolonged course, can result in a clinical constellation of findings that makes one think of connective tissue disease (CTD). Autoantibodies such as antinuclear antibodies (ANA) have been detected in about 30% of patients with iMCD, as these can increase in the course of polyclonal hypergammaglobulinemia and do not rule out the presence of iMCD [3]. Conversely, up to 59% of patients with SLE may have lymphadenopathy [20]. If pleural effusion, anemia, and thrombocytopenia are still present, the diagnosis of SLE seems obvious [21], especially since only skin manifestations such as eruptive hemangiomatoses or purple papules are atypical for CTD as clinical criteria for CD. The questioning of a pre-diagnosed CTD or histological re-evaluation is particularly indicated if the clinical and serological course does not match the SLE diagnosis, or if the patient is male or of advanced age at the time of initial manifestation or has not responded to previous therapy. SLE, on the other hand, is more likely with typical or histologically confirmed skin and mucosal manifestations, neuropsychiatric involvement, arthritis, glomerulonephritis, detection of specific antibodies (such as homogeneous ANA and corresponding anti-dsDNA or anti-Sm antibodies), and complement consumption.

The diagnostic differentiation from Sjögren’s syndrome (SS) is successful in the case of sicca symptoms known for many years, histology, and with sonography of the large salivary glands, as well as with the detection of typical autoantibodies against SSA (Ro)/SSB (La). There have been single cases reported in which the diagnosis of iMCD was complicated by an accompanying SS [22].

In contrast to CD, both CTDs are usually associated with comparatively low/normal CRP.

## IgG 4-associated disease

Another important differential diagnosis in rheumatological practice is represented by iMCD in IgG 4-related disease (IgG 4-RD). The distinction is also important because the treatment of the two entities differs considerably. Lymph node involvement is obligatory in iMCD but is also observed in IgG 4-RD—with the potential formation of large abdominal masses in both diseases. Infiltration of the lung or kidney parenchyma [23, 24] has also been described in both iMCD and IgG 4-RD, although it is not possible to distinguish between them on the basis of imaging alone. Both diseases may be associated with hypergammaglobulinemia and an increase in the concentration of IgG 4 in serum as well as in tissue biopsy. On average, a significantly higher IgG 4/IgG ratio was observed in patients with IgG 4-RD than in patients with iMCD. In addition to elevated IgG and IgM levels, persistently elevated CRP levels have been proposed as a serological exclusion criterium for IgG 4-RD [25].

In the absence of lymph node involvement, iMCD can be practically ruled out, and the involvement of orbit, salivary glands, and pancreas also speaks against this diagnosis. Splenomegaly, on the other hand, is atypical for IgG 4-RD. In iMCD, elevated IL‑6 concentrations are often found [26] but also higher CRP, IgA, and IgM levels as well as frequent fever. In contrast, the immunopathogenesis of IgG 4-RD is based on IL-4-driven activation of Th2 cells, which is why atopic diathesis is observed more frequently here (Table 3; [27]). Histologically characteristic of IgG 4-RD in the lymph node is an expansion of the germinal centers, and vertebral or spoke wheel-like fibrosis in parenchymal organs. In principle, in the case of a poor response to glucocorticoids [28], it is also advisable to fundamentally review the IgG 4-RD diagnosis and to question whether the symptom constellation might be more compatible with another immunological systemic disease such as iMCD.

**Table 3:** IgG 4-RD- and iMCD-associated characteristics (exceptions are possible): combined consideration of clinical, serological, and pathological findings plus response to a treatment attempt is recommended [25, 27]

Characteristics	lgG4-RD	iMCD
*Clinical*		
History of atopy	Frequent	Normal
Involvement of exocrine glands	Frequent	Rarely
Lymph node involvement	Occasionally	Always
*Biomarkers*		
Hemoglobin	Normal	Low
Platelet count	Normal	High
Albumin	Normal	Low
lgG4:lgG ratio^a^	High	Normal
IgA	Normal	High
IgM	Normal	Hoch
IL‑6	Normal	Hoch
*Histology*		
Enlarged germinal centers	Frequent	Occasionally
Proliferation of mature plasma cells	Rarely	Frequent
Hemosiderin deposition	Rarely	Frequent
lgA+ cells	Rarely	Pronounced

^a^An lgG4:lgG ratio > 40% is considered pathological in lgG4-RD [25]

## Adult-onset Still’s disease

Sharply increased acute-phase proteins, leukocytosis, fever, swallowing difficulties, and enlarged cervical lymph nodes also suggest adult-onset Still’s disease (AOSD). AOSD is characterized by bicyclic fever attacks in the morning and evening as well as a transient small-spotted confluent skin rash, especially during a fever episode. For the diagnosis of AOSD, the Yamaguchi criteria can be used [1], which can also be fulfilled without the typical skin rash (Still’s syndrome). However, before their use, infections, hematological diseases, and other rheumatological diseases must be excluded. In particular, in the absence of the (characteristic) Still’s rash, further differential diagnoses should be considered. In terms of laboratory tests, we expect a pronounced hyperferritinemia in AOSD in acute episodes, which is not found in CD. In the case of persistent swelling of the cervical lymph nodes over 4 weeks or significant enlargement of single or multiple lymph nodes to over 1 cm, it is recommended that a well-accessible lymph node be removed as completely as possible.

## AOSD-like disorders

From a histopathological perspective, the differentiation from infection-related diseases can be challenging. Particularly in younger patients, the possible presence of infectious mononucleosis (initial Epstein–Barr virus infection) should also be considered, with the lymphocytic types of stimulus in the peripheral blood smear, also referred to as virocytes, pointing the way forward. Confirmation can be made by EBV DNA detection. Chronically active EBV infection may indicate a primary immunodeficiency (PIK3C, CD27, or CD70 deficiency; mutations in *SH2D1A* or *XIAP*) [25, 26, 29, 30]. In elderly male patients without leukocytosis but with macrocytic anemia and further evidence of a dysplastic disorder of hematopoiesis (myelodysplastic syndrome of the chronic myelomonocytic leukemia type), VEXAS syndrome should also be excluded. This is a heterogeneous inflammatory disease caused by acquired somatic mutation of the *UBA1* gene on the X chromosome and is usually associated with MDS-like changes in blood counts. Phenotypically, VEXAS syndrome may be reminiscent of pseudo-AOSD disease [31]. Skin symptoms can also occur here, but these are not to be confused with Still’s exanthema, as well as manifestations such as polychondritis or vasculitis [23]. In addition, paraneoplastic syndromes can mimic AOSD-like disease [32].

## Macrophage-activation syndrome and HLH

Secondary macrophage-activation syndrome (MAS) can be observed in a subgroup of patients with AOSD and in other highly inflammatory systemic diseases. Primary MAS can occur in early childhood (referred to as hemophagocytic lymphohistiocytosis [HLH]), with the possibility of high levels of ferritin, AST, and LDH; increasing uni-, bi-, or trilinear cytopenia; and episodes of fever and swelling of the lymph nodes. Hemophagocytosis can be detected in 50% of the smears of bone marrow aspirates. The sensitivity and specificity of the MAS-HLH diagnosis can be determined with the HScore [33].

## Treatment of CD

As iMCD is potentially life threatening, immediate initiation of treatment is indicated. Based on data from a randomized clinical trial, the anti-IL‑6 monoclonal antibody siltuximab is recommended by international guidelines as the preferred first-line therapy and is the only EMA- and FDA-approved agent for iMCD [5, 34]. Approval was based on a randomized trial in which 79 patients with iMCD were treated with either siltuximab or placebo every 3 weeks [34]. The primary endpoint was a response sustained for at least 18 weeks, as measured by a reduction in tumor size and improvement in clinical symptoms. While no response was observed in the placebo group, durable tumor and symptom response was 34% in the verum group. A durable symptomatic response was observed in 57% in the siltuximab arm [34]. Patients usually respond after 3 to 4 doses (laboratory, clinical), and a decrease in lymphadenopathy is observed later in our experience (initial controls every 3 months by imaging). In most cases, IL-6-inibitory therapy must be continued permanently [5, 35]. Long-term data over 6 years are now available on the tolerability and efficacy of siltuximab [32]. Especially at the beginning, a combination with corticosteroids should be used (prednisone 1–2 mg/kgKG), which are phased out over 4 to 6 weeks [35]. If siltuximab is not available, tocilizumab can be an alternative treatment option [36, 37]. In patients with mild disease and minor clinical symptoms, rituximab plus steroids can initially be considered [35]. Depending on the clinical features, the international guideline describes the use of rituximab in combination with immunomodulators or polychemotherapy for the second line [36]. Accordingly, in the event of treatment failure or in the case of highly active disease, a decision should also be made quickly, together with the hemato-oncologists, regarding potential polychemotherapy (CHOP ± R, VDTPACE, CVAD ± R). For patients who do not respond to anti-IL‑6 therapy or who are in the relapsed situation, treatment recommendations are usually based on evidence from case series or individual case reports [38]. In recent years, activation of the mTOR kinase and JAK/STAT signaling pathways has been shown in iMCD, providing potential future therapeutic targets [39]. Imaging techniques (e.g., FDG-PET/CT) can be used to assess treatment response or monitor disease progression in addition to laboratory chemical parameters [40].

## Conclusion

- In contrast to unicentric Castleman’s disease (UCD), idiopathic multicentric CD (iMCD) is not a localized, surgically treatable lymph node hyperplasia: iMCD is a serious, potentially fatal, systemically progressive disease.
- The rarity of the disease causes the limited availability of data on the frequency of iMCD, but the incidence and prevalence are, in principle, likely to be underestimated: due to the heterogeneous clinical presentation and courses as well as overlaps with malignant, infectious, or autoimmune diseases, there is a high probability of misdiagnosed or undetected cases.
- The spectrum of immune-mediated diseases that are important in the differential diagnosis ranges from SLE/SS, RA/JIA, adult-onset Still’s disease (AOSD), AOSD-like diseases, ALPS, IgG 4-associated diseases, to HLH and VEXAS syndrome.
- From a pathologist’s perspective, it is crucial to think about the differential diagnosis of iMCD early on. For optimal histological assessment, a complete lymph node is required; a puncture of the lymph node is not sufficient.
- Siltuximab is an approved drug therapy for iMCD. The principle of treatment is to inhibit the IL‑6 signaling pathway. Alternatively, other IL-6 inhibitors or, in mild cases, rituximab could be used as an individual attempt at treatment.

## Declarations

For this article no studies with human participants or animals were performed by any of the authors. All studies mentioned were in accordance with the ethical standards indicated in each case.

The supplement containing this article is not sponsored by industry.
